# The Cognitive Remediation in Bipolar (CRiB) pilot study: study protocol for a randomised controlled trial

**DOI:** 10.1186/s13063-016-1472-4

**Published:** 2016-07-29

**Authors:** Rebecca Strawbridge, Jessica Fish, Rozmin Halari, John Hodsoll, Clare Reeder, Karine Macritchie, Paul McCrone, Til Wykes, Allan H. Young

**Affiliations:** 1 Centre for Affective Disorders, Institute of Psychiatry, Psychology & Neuroscience, King’s College London, De Crespigny Park, Denmark Hill, London, SE5 8AF UK; 2 The NIHR Biomedical Research Centre at the South London and Maudsley NHS Foundation Trust and the Institute of Psychiatry, Psychology and Neuroscience, King’s College London, London, UK; 3 Oliver Zangwill Centre for Neuropsychological Rehabilitation, Cambridgeshire Community Services NHS Trust, Princess of Wales Hospital, Lynn Road, Ely, CB6 1DN UK; 4 OPTIMA Mood Disorders Service, Lambeth Hospital, South London and Maudsley NHS Foundation Trust, London, SW9 9NU UK; 5 Department of Biostatistics, King’s Clinical Trials Unit, King’s College London, De Crespigny Park, Denmark Hill, London, SE5 8AF UK; 6 Department of Psychology, Institute of Psychiatry, Psychology & Neuroscience, King’s College London, De Crespigny Park, Denmark Hill, London, SE5 8AF UK; 7 Department of Health Services and Population Research, King’s Health Economics, Institute of Psychiatry, Psychology & Neuroscience, King’s College London, De Crespigny Park, Denmark Hill, London, SE5 8AF UK

**Keywords:** Bipolar disorder, Cognitive remediation therapy, Randomised controlled trial, Trial protocol

## Abstract

**Background:**

People with bipolar disorder often show difficulties with cognitive functioning, and though these difficulties are identified as important targets for intervention, few treatment options are available. Preliminary evidence suggests that cognitive remediation therapy (a psychological treatment proven beneficial for people diagnosed as having schizophrenia) is helpful for people with bipolar disorders. We are conducting a pilot trial to determine whether individual, computerised, cognitive remediation therapy (CRT) for people with bipolar disorder 1) increases cognitive function; 2) improves global functioning, goal attainment and mood symptoms; 3) is acceptable and feasible for participants; and 4) can be addressed in a comprehensive, larger, randomised, controlled trial.

**Methods/design:**

The study is designed as a two-arm, randomised, controlled trial comparing cognitive remediation therapy with treatment-as-usual (TAU) for euthymic bipolar patients. Participants are eligible to take part if aged between 18 and 65 with a diagnosis of bipolar disorder (type I) and currently in euthymic state, and no neurological, substance or personality disorder diagnoses. Sixty participants will be recruited (mainly through secondary and tertiary care) and will be block-randomised to receive either treatment-as-usual alone or in addition to a 12-week course of cognitive remediation therapy totalling 20–40 therapy hours. The intervention will comprise regular sessions with a therapist and computer-based training. Research assessments will take place before and after the intervention period and at a 12-week follow-up, and will include evaluation of neuropsychological, symptom-related, demographic and social factors, as well as collecting qualitative data regarding CRT expectations and satisfaction. Intention-to-treat analyses will examine the efficacy of cognitive remediation therapy primarily on cognition and additionally on functioning, quality of life and mood symptoms. Furthermore, we will examine the acceptability of CRT and undertake a preliminary health economics analysis to ascertain the cost of delivering the intervention.

**Discussion:**

The results of this trial will provide valuable information about whether cognitive remediation therapy may be beneficial for people diagnosed with bipolar disorder in a euthymic state.

**Trial Registration:**

ISRCTN registry, ISRCTN32290525. Registered on 2 March 2016

## Background

Bipolar disorder is one the most disabling health-related conditions, contributing at least 2 % to the total disability adjusted life-years of non-communicable diseases worldwide in 2005 [1]. It typically first occurs at the beginning of adult life, when life-long relationship and occupational trajectories are established, potentially causing serious and long-lasting social and functional impairment. The cost to society is also considerable, both in terms of the lost productivity of individuals and in the direct costs related to provision of health care [2]. The provision of secondary preventative treatment would reduce the personal burden of the illness, and fewer future hospitalisations would provide a significant cost saving to health services. While research indicates that overall individuals with psychiatric diagnoses show better recovery when pharmacological and psychological treatments are provided concomitantly [3], further research is required to develop and investigate useful psychological interventions that are cost-effective and could be provided routinely in health services for bipolar disorder.

One potential target for therapy is cognition: bipolar disorder is associated with widespread cognitive deficits (perhaps most notably in executive function) that can persist even during periods of recovery and are associated with poorer levels of functioning [4]. Findings are consistent with a pattern of cognitive deterioration as the number and severity of the episodes increase, and cognitive impairment is a factor that may predict increased recurrence of episodes [5]. Therefore, an improvement in cognition might enhance not only short-term function and quality of life (possibly facilitated by meta-cognition) but potentially also the overall course of illness. This pattern of findings in bipolar disorder is similar to schizophrenia, for which a new psychological treatment targeting cognition has shown promising results: cognitive remediation therapy (CRT). CRT aims to target fundamental cognitive processes, including attention and memory, but also executive functioning which requires the combination of basic cognitions and metacognitive skills to aid the transfer into everyday practice of improved cognition to enhance quality of life [6]. In people with diagnoses of schizophrenia, CRT has demonstrated improvements in the realms of cognitive, work and social functioning, quality of life and severity of symptoms experienced [7–9].

In mood disorders, a meta-analysis of CRT trials including patients with various affective disorders has demonstrated a beneficial effect of CRT on cognitive performance [10], although no trials included a purely bipolar sample. More recently, a pilot trial using a functional targeted therapy that included cognitive remediation identified benefits to the quality of life for people with bipolar disorder [11, 12].

CIRCuiTs is a partly computerised CRT intervention, developed from a paper-and-pencil version of the therapy and building upon the existing CRT literature. Using the program, clients are taught cognitive strategies to help them improve their thinking skills (e.g. memory, concentration and planning), and the therapist facilitates reflection around how these strategies can be applied to meet their everyday goals (e.g. shopping, looking for work) including the application of metacognitive strategies. CIRCuiTs has demonstrated high levels of acceptability, usability and comprehensibility to participants, with high levels of satisfaction with the experience reported [13].

We anticipate that this intervention may facilitate optimisation of the management of bipolar disorder through improvements in cognitive functioning in combination with the development of metacognitive skills and elements focusing on transfer to everyday life. As such, a pilot randomised trial is being conducted to investigate whether a cost-effective but intensive cognitive remediation therapy (CIRCuiTs) is a viable treatment for bipolar disorder.

### Objectives

The overarching aim of this trial is to determine whether CRT can serve as a new, evidence-based treatment for bipolar disorder. In order to achieve this, the following research questions are being investigated as primary objectives, as they are all fundamental pre-requisites of future examination of CRT for bipolar disorder:

1. Does CRT improve overall cognition (specifically learning, verbal and working memory, intellectual functioning, attention and executive functions)?
2. Does CRT provide further, specific benefits to participants (i.e. everyday functioning, mood symptoms and achievement of self-defined goals)?
3. Do people with bipolar disorder feel that CRT is beneficial and acceptable?
4. Can sufficient patients be recruited to conduct a larger, more comprehensive RCT?

Potential moderators or mediators for the benefits of CRT will additionally be explored on a secondary basis.

## Methods/design

The study is being conducted in accordance with CONSORT and SPIRIT guidelines. The CRiB study was approved by City Road & Hampstead NHS Research Ethics Committee on 16/10/2015 (reference 15/LO/1557).

### Trial design

**Fig. 1:**
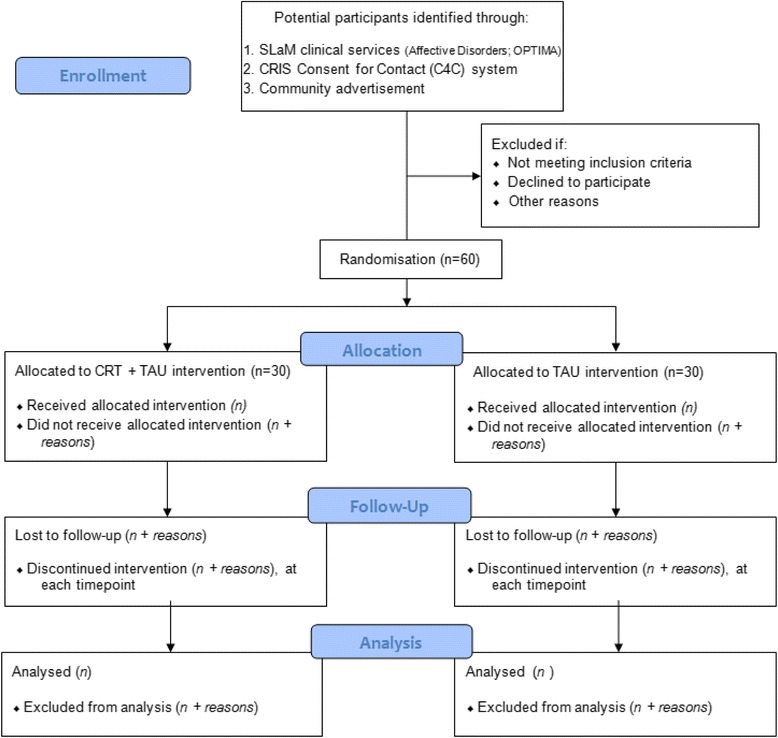
CONSORT flow diagram. The flow chart depicts participant progression through the study from initial enrolment through allocation, follow-up and finally analyses of their data stages

See Fig. 1 for the study flow chart. This is a pilot and feasibility randomised controlled trial, which comprises a 12-week intervention period followed by a 12-week follow-up period. Randomisation will ensure a 1:1 allocation ratio of participants to either Cognitive Remediation Therapy (CRT) in addition to Treatment-As-Usual (*n* = 30) or TAU alone (*n* = 30) for the intervention period. Measures will assess the outcomes before (baseline; week 0), after (week 13) the intervention period, and after the follow-up period (week 25). Two self-report symptom measures of depression and mania will be completed by all participants at weeks 1, 4, 8 and 12 during the intervention period to monitor wellbeing.

### Setting

The study will take place in one of two clinical academic settings in South London: The Clinical Research Facility (CRF), King’s College Hospital, or the OPTIMA mood disorders clinic, Lambeth Hospital, both South London and Maudsley NHS Foundation Trust (SLaM; London, UK). Both are quiet and private spaces that the participant can select from at their convenience but for each participant will be consistent between all assessment sessions.

### Participants

Thirty participants will be assigned to the intervention arm (CRT with TAU) and 30 to the control arm (TAU alone); this sample size is recommended for pilot trials [14] to estimate efficacy by corroborating effect sizes for cognitive and functional outcomes. We anticipate recruiting approximately half of the sample from secondary/tertiary care and half from the community (approximately 15 from each recruitment strand per group). Community recruitment will comprise public advertisements using methods that have previously been successful [15]. Participants will be recruited if fully informed and willing to participate and if they meet the following inclusion criteria: aged 18–65, have a confirmed DSM-V diagnosis of bipolar I disorder (using the Mini International Neuropsychiatric Interview [16]), and be in a euthymic state at two time points 1 week apart, defined by scores of < 8 on the Hamilton Rating Scale for Depression and Young Mania Rating Scale (standardised cut-off scores for euthymia [17] on both occasions. No constraints exist based on sex or prior treatment, but fluency in English and the ability to use a computer are required (assessed verbally). Furthermore, we will exclude potential participants if they have evidence of a substantial neurological, neurodegenerative, substance-use or current diagnosis of personality disorder (assessed using standardised measures). Both women who are pre- and post-menopausal will be included; menopausal status and current phase of menstrual cycle at each assessment time point will be reported verbally and recorded. This will also be controlled for in analyses due to reliable findings of cognitive variations across the menstrual cycle [18].

Participants will be selected from services within South London and Maudsley NHS Trust (the national Affective Disorders Service and the OPTIMA Mood Disorders Programme), by identification of potentially eligible individuals through the Consent for Contact (C4C) SLaM initiative [19], which allows approved researchers access to a computerised retrieval system to rapidly screen limited, relevant case notes of consenting service users and by community advertisements and a research webpage.

### Procedures

#### Pre-inclusion

Following provision of study information and verbal consent for screening, the initial screening interview (Screen A) will involve questions about demographics (age, received diagnoses), a diagnostic assessment and researcher-rated and self-report measures of depressive and manic symptoms. If not excluded so far, a second screening (Screen B) will take place 1 week later to validate stable asymptomatic state. Prior to inclusion, bipolar disorder diagnoses will be validated by a practising psychiatrist from the study team, by verbally discussing the scoring of the MINI assessment with the assessor and requesting further information if required).

#### Randomisation

Following fully informed written consent, participants will be randomised to one of the two treatment arms (TAU or TAU + CRT). Randomisation is facilitated by the King’s College Clinical Trials Unit independent web-based system [20], holding the details needed for randomisation (treatment program, date of birth, and unique identity number). Trial participants will be randomised between the two parallel groups in a 1:1 allocation (30 participants per treatment group). A stratified block randomisation will allocate participants to treatment groups, with an equal number of participants in each stratification group (intensive-secondary or tertiary care; standard secondary care) using an initial random allocation sequence (n cases). All randomisation details will be locked following treatment allocation. The lead researcher (RS) will not be blinded to treatment allocation but the researcher conducting research assessments and trial statistician will be blind.

#### Treatment as usual

All 60 participants will continue receiving treatment as usual; the study does not affect concomitant interventions in any way. However, we will measure service use, so all current treatments for all patients are recorded. TAU is not expected to affect the outcome measures or systematically differ between participants, but intensity of current treatments is stratified between intervention arms.

#### CRT intervention

CRT will be delivered over the course of a 12-week period, flexibly utilising a combination of formats (i.e., face-to-face, telephone, drop-in sessions and individual practice), amounting to approximately 30–40 hours of CRT. Face-to-face sessions (up to 1 hour long) will be held at least once per week, but participants will be encouraged to attend up to three sessions per week if feasible; an alternative arrangement of twice-weekly telephone sessions (in addition to one face-to-face session) will be offered. Additionally, all individuals will be provided unlimited access to use the program in their own time, for practising independently as they wish, with an option to receive reminder text-messages that can be sent to participants up to twice per week. This intervention aims are tailored to individual needs, using a format beneficial for themselves specifically. Where possible, and only with the participants’ agreement at each session, therapy meetings will be video-recorded or auditory-only recorded; this will assist with ensuring therapist fidelity and optimising the intervention for future use. CRT is considered a safe therapy, unlikely to have an impact on risky behaviours, and the participants should not be experiencing symptoms during the trial. Though we perceive this not to be a high-risk study, the participants are considered a vulnerable population, and a small risk exists that they will not engage positively with, or more importantly, will have a negative emotional reaction to the intervention (e.g. frustration may be experienced upon failure to complete a particular task). Despite existing evidence indicating that people find CRT helpful and acceptable, we will ensure that CRT therapists are comprehensively trained and prepared to manage such situations (using supportive and solution-focused approaches). Effort will be put into avoiding such circumstances by following standardised protocols which researchers will be trained in for adverse situations. We emphasise that the intervention is delivered using positive methods and has reliably elicited positive feedback from patients during previous trials. CRT therapists will be psychologists (to post-graduate-level study at minimum), who have received specific training from experts in the intervention, on CRT and the CIRCuiTs program specifically. They receive ongoing supervision from experienced CRT practitioners. Processes are in place to deal with any safety issues that include ongoing participant monitoring throughout the intervention period; completion of self-report symptom measures on a monthly basis, and prompts from a researcher to safeguard completion and scoring. In any instances where scores exceed the standardised cut-offs for depression or mania, individuals will receive a call from a researcher (or a therapist if concerns persist). If necessary, a therapist or clinician and participants will discuss and agree whether there is a need for trial withdrawal. Additional concerns about safety and wellbeing will be conveyed to the participant’s contact healthcare professional. A copy of the consent form alongside a letter of notification and information sheet is sent to nominated healthcare professional for all participants. Trial participation (and whether CRT is being undertaken) is to be recorded on patient’s health records as the trial affects clinical care and may be important for treatment-related decisions. A data management plan (in adherence with the Research Governance Framework) has been developed to ensure protocols for quality assured monitoring and safety are maintained during the trial. Data monitoring support is also accessible through the trial steering committee and trial specific working manual.

### Measures

See Table 1 for a summary of study measures at each time point.

**Table 1:** Study measures and time points

Concept	Measure	Screen A	Screen B	Week 0	Week 1	Week 4	Week 8	Week 12	Week 13	Week 25
Cognitive	Wechsler Adult Intelligence Scale (WAIS)			X					X	X
	Wechsler Abbreviated Scale of Intelligence (WASI)			X					X	X
	Wechsler Memory Scale (WMS)			X					X	X
	Delis-Kaplan Executive Function System (DKEFS)			X					X	X
	Hotel Test			X					X	X
	Test of Premorbid Functioning (TOPF)			X						
	Perceived Deficits Questionnaire (PDQ)			X					X	X
Functional	Functional Assessment Short Test (FAST)			X					X	X
	Goal-attainment Scale (GAS)			X					X	X
Mood-related	Toronto Alexithymia Scale (TAS-20)			X					X	X
	Hamilton Rating Scale for Depression (HAMD)	X	X	X					X	X
	Young Mania Rating Scale (YMRS)	X	X	X					X	X
	Inventory of Depressive Symptomatology (IDS)	X	X	X	X	X	X	X	X	X
	Altman Self-rating Mania scale (ASRM)	X	X	X	X	X	X	X	X	X
	Hamilton Anxiety Rating Scale (HAM-A)			X						
Psycho-social	Childhood Trauma Questionnaire (CTQ)			X						
	Structured Clinical Interview for DSM (axisII), BPD scale (SCID-BPD)			X						
	Standardised Assessment of Personality – Abbreviated Scale (SAPAS)			X						
	Trait Self-description Inventory (TSDI) of personality			X						
Other	Qualitative questions			X					X	
	Client Service Receipt Inventory (CSRI)		X						X	X
	WHO quality of life measure (EQ-5D)		X						X	X
	Mini Neuropsychiatric Interview (MINI)	X								
	Montreal Cognitive Assessment; (MoCA)	X								
	Demographic information			X					X	X

Week 0 = baseline assessment, Week 13 = post-intervention assessment, Week 25 = follow-up assessment

#### Primary outcomes

The putative primary outcome measure is the digit symbol coding test from the Wechsler Adult Intelligence Scales 4th Edition (WAIS [21], akin to the digit symbol-substitution test (DSST)), measured at baseline (week 0), at post-intervention (week 13) and follow-up assessments (week 25). This was selected as the measure which has been identified widely as sensitive to change and representative of cognition, measuring domains including but not limited to visuospatial processing, attention control and switching, associative learning, executive control and short-term memory [22, 23].

Also measured at Weeks 0, 13 and 25, the remaining cognitive outcomes assess: current IQ performance, measured in the WASI-II test (two-subtest version of the Wechsler Abbreviated Scale of Intelligence 2nd Edition [24]; verbal memory assessed in the verbal paired associates I and II tasks from the Wechsler Memory Scales 4th Edition [25] (WMS); digital memory and reasoning measured in the digit span and symbol search tasks from the WAIS (Wechsler Adult Intelligence Scales 4th Edition [21]); verbal fluency assessed in the Delis-Kaplan Executive Function System (DKEFS [26]); executive function, from the Hotel test of planning and multi-tasking abilities [27] revised version (personal communication JF, Dr Tom Manly). A composite score (comprising all of the above cognitive outcomes) of cognition will also be calculated for feasibility and the acceptability of the intervention (measured using numbers completing CRT and number of hours undertaken) is also a primary outcome, assessing objective 3. Perceived cognitive impairments are evaluated using the Perceived Deficits Questionnaire (PDQ [28]) at weeks 0, 13 and 25.

In addition, at the three main assessment time points (weeks 0, 13 and 25), the additional outcomes are measured: The Functional Assessment Short Test (FAST [29]) calculates everyday functioning; the Goal Attainment Scale (GAS), a personalised measure of goal attainment [30]; and experiences of alexithymia (assessed using the Toronto Alexithymia Scale; TAS-20 [31]. Researcher-rated severity of depressive (Hamilton Rating Scale for Depression; HAMD [32]) and manic (Young Mania Rating Scale; YMRS [33]) symptoms are conducted to establish euthymia at screening (both A and B) and at the three assessment time points (weeks 0, 13 and 25). Subjective symptom assessments are additionally conducted at all study time points (0, 1, 4, 8, 12, 13 and 25 weeks) using the Inventory of Depressive Symptoms (IDS [34]) and Altman Self-Rated Mania (ASRM [35]) scales.

Before (week 0) and after the intervention (week 13), qualitative questionnaires will measure the feasibility and satisfaction with Cognitive Remediation Therapy [36].

Extent of current health service-usage and health-related quality of life are assessed using an adapted version of the Client Service Receipt Inventory (CSRI [37]) and the EQ-5D measure [38]) respectively, at the second screening session (Screen B) and then after treatment (week 13) and follow-up (week 25). These measures are used also to calculate the estimated cost of providing CRT (alongside data recorded from CRT, e.g. number of therapist hours).

Attendance to face-to-face and telephone sessions, as well as amount of time spent individually practising CRT will be monitored; the CIRCuiTs program records extensive data regarding usage automatically.

#### Secondary outcomes

Approximate premorbid IQ is measured using the Test of Premorbid Function (TOPF [39]) at baseline (week 0). Also at the baseline assessment only (week 0), anxiety levels are measured using the Hamilton Anxiety rating scale (HAM-A [40]), history of childhood trauma using the Childhood Trauma Questionnaire (CTQ [41]), personality disorder traits (using the SCID-BPD; Structured Clinical Interview for DSM-IV disorders, borderline scale [42] and SAPAS; the Standardised Assessment of Personality–Abbreviated Scale [43]) and general personality traits (assessed using the TDSI; The Trait Self-Description Inventory [44]).

#### Other measures

At Screen A, the diagnostic assessment includes the MINI (Mini International Neuropsychiatric Interview [16] structured assessment for common mental disorders; the bipolar disorder, substance use, and personality disorder sections to be completed first to determine eligibility, and the MoCA (Montreal Cognitive Assessment [45]) which screens for symptoms of dementia (for inclusion total score must be below 26). Demographic information collected (at Screen A and week 0) includes data regarding age, employment, physical health and recent life events. All regular medications of each participant will be recorded and any changes in medication will be noted at the post-intervention and 12-week follow-up sessions. We will carefully monitor adverse events and patient safety at every time point (Weeks 0, 1, 4, 8, 12, 13 and 25), and any concerns in this regard will be promptly taken to the Lead PI and Steering Committee.

### Statistics

Analyses will be carried out by the trial statistician. The main purpose of the statistical analyses is to estimate trial and intervention feasibility parameters. Thus, we will use largely descriptive statistics with inferential statistics used to generate respective confidence intervals and effect sizes. The analyses can be split into three aims: 1) estimation of parameters that evaluate trial feasibility (recruitment rates, randomisation rates and attrition rates), to meet objective 4; 2) estimation of parameters that measure intervention feasibility (adherence, treatment fidelity and patient satisfaction) in accordance with objective 3; and 3) estimates of intervention effect sizes (mean differences and standard deviations) in terms of potential primary outcomes of future trials or variables that are targeted by the therapy (putative mediators of future treatment effects), addressing objectives 1 and 2. All statistical analyses will adopt the intention-to-treat (ITT) principle and will be conducted after data collection has been completed.

#### Trial feasibility (objective 4) and intervention feasibility (objective 3) parameters

The analysis will firstly consist of descriptive statistics to assess the rate of consent of eligible patients, the acceptability of CRT to the patient population, treatment fidelity, deviations from study protocol and study retention. Participant demographic and clinical characteristics will also be summarised at baseline. Patterns of missing data or nonattendance will be described for the outcome at measurement time-points and according to therapy session, along with reasons for drop-out. As appropriate, the statistics derived will be means and standard deviations or medians, minimum values, maximum values and interquartile range for continuous measures and proportions for ordinal or multinomial categorical measures and binary coded measures.

#### Estimates for effect sizes for CRT versus TAU (objectives 1 and 2)

To assess improvement in cognitive and functioning domains, estimates of treatment effect sizes will be obtained using linear mixed models. These analyses will provide the treatment effect estimate on each outcome at 13 and 25 weeks. The linear mixed model will contain post-treatment measures of the outcome at the two follow-up time points, i.e. 13 and 25 weeks as the dependent variables with fixed effects of trial arm, baseline measure of outcome, time and a time point by trial arm interaction to allow treatment effects to differ at 13 and 25 weeks. Baseline measures of the primary outcomes are included as they are potential predictors of future outcome and thus should help us to gain precision for effect estimates of interest. A random effect for participant will be entered in to the model to account for correlations between the two repeated measures per participant. In addition, to account for the correlation of level-1 residuals across time, an exchangeable residual covariance structure will be included in the model. We will also consider changes in cognitive variables as mediators of improvements in functional outcomes (FAST). The aim here is to derive an effect size and inferential statistics will not be reported.

With respect to missing data, linear mixed models will be estimated using maximum likelihood (ML). Such an approach provides valid inferences under the assumption that the missing data mechanism is ignorable (or missing at random, MAR). Here, this means that explanatory variables included in the model can predict missingness as can earlier observed values of the outcome. We will include baseline predictors of drop-out as explanatory variables in the linear mixed model to make the MAR assumption more realistic. However, given the small dataset the number of explanatory variables to be included will be necessarily small. Finally, Sensitivity analysis will be used to assess the robustness of the conclusions to missing outcome data in which missing values will be imputed according to various plausible scenarios, e.g. participants with missing data showing no improvement in cognitive outcomes.

#### Economic analyses

Service use measured with the Client Service Receipt Inventory will be combined with appropriate unit costs [46]). The costs of the intervention, which will be based on the resources required to deliver it (staff time and overheads) and activity levels, will be added to these. The EQ5D will be used to derive quality-adjusted life years. These will be combined with the costs and comparisons made between the two groups. Uncertainty will be addressed using cost-effectiveness planes derived though bootstrapped resamples.

## Discussion

CRT has the potential to increase cognitive function and for improvements to translate to quality of everyday life and social and occupational functioning, as well as possibly to prevent the onset of mood symptoms. Our findings should indicate whether a partly computerised, 12-week course of cognitive remediation therapy can improve cognition above treatment-as-usual.

### Strengths

This study has been designed and is being conducted in compliance with the principles of the Declaration of Helsinki (1996), the principles of Good Clinical Practice (GCP) and in accordance with all applicable regulatory requirements including but not limited to the Research Governance Framework and the Mental Capacity Act (2005). These actions will maximise validity, participant safety and scientific integrity as well as reduce bias. Additionally during the planning stages we sought review of the scientific quality of the study from a service user panel (NIHR/MHRN’s FAST-R - Feasibility and Support to Timely recruitment for Research - service) and informally by experienced academics and clinicians prior to two stringent peer review processes for funding application. The study was judged to be well-designed by a strong team, and to be worthwhile in terms of putative future implications. Trial Steering Committee monitoring (which includes all study investigators, individuals independent from the study team and service user representatives), regular and experienced supervision for therapists, and ongoing support and training for those undertaking research assessments will ensure that quality is maximally maintained throughout the trial.

### Limitations

Participants will be recruited via a number of routes, and although this may increase the representativeness of people with bipolar type I disorder, it may also increase heterogeneity of our findings. The flexibility of the CRT intervention (in terms of different modes of provision and aspects focused on) may also increase heterogeneity. Due to participant burden and resources, we are not able to account for all potential confounders. In addition, as we are not excluding participants based on cognitive deficits, it is possible that some of our participants might have high functioning and therefore not show improvements with CRT (something important to be addressed in future studies). The sample size for this pilot trial is relatively small to detect differences of the domains being measured and provides a comparison of CRT with treatment as usual care alone. The results will inform a more comprehensive RCT comparing a CRT intervention with an active placebo condition, to establish the effects of CRT for a group of people diagnosed with bipolar disorder controlling for non-therapeutic but potentially important factors (e.g. cognitive task-specific improvements and time engagements).

This publication of our protocol aims to maximise reproducibility and transparency of this pilot trial, which is investigating the effects of CRT for people with bipolar disorder; our findings will indicate whether this accessible program provides benefits to this population over treatment-as-usual, which would represent a vital advance in psychological care for asymptomatic bipolar disorder.

## Trial status

The trial is ongoing. Recruitment commenced in February 2016.

## Abbreviations

C4C, Consent for Contact—SlaM service to facilitate patient recruitment into research; CIRCuiTs, Computerised program for cognitive Remediation Therapy; CRF, clinical research facility; CRiB, Cognitive Remediation in Bipolar study; CRT, cognitive remediation therapy; DSM-IV, Diagnostic & Statistical Manual for Mental Disorders version IV; OPTIMA, Specialist clinical service in SLaM for affective disorders; RCT, randomised control trial; SLaM, South London & Maudsley NHS Foundation Trust; TAU, treatment as usual
